# Supplementary material for: The stabilized Pol31–Pol3 interface counteracts Pol32 ablation with differential effects on repair
Source: Life Sci Alliance. 2021 Jul 5;4(9):e202101138. doi: 10.26508/lsa.202101138 (PMC8321694; doi:10.26508/lsa.202101138)
Supplement: Supplementary file 1 [file LSA-2021-01138_TableS1.docx]

**Table S1: Yeast strains used in this study.** The complete genotype of budding yeast strains used in this study are listed with the source of the strain.

| Strain | Genotype | Plasmid | Source |
| --- | --- | --- | --- |
| *GA-4619* | (KSC106) *MATa, ade1, his2, trp1, ura3, leu2* |  | (K. Sugimoto) |
| *GA-4620* | GA-4619 with *pol31::KanMX4* | pRS316-POL31 | (Davoodi et al., 2006) |
|  | KSC106 with *pol31::KanMX4, pol31-D297A* | pPOL31-D297 | (Davoodi et  al., 2006) |
| *GA-9784* | GA-4619 with *pol31::KanMX4, pol31-EF463AA* | pPOL31-EF463 | this study |
| *GA-9782* | GA-4619 with *pol31::KanMX4, pol31-W417A* | pPOL31-W417 | this study |
| *GA-4761* | GA-4619 with *pol32::hphMX* |  | this study |
| *GA-4639* | GA-4619 with *pol31::KanMX4, pol32::hphMX, pol31-EF463AA* | pPOL31-EF463 | this study |
| *GA-4636* | GA-4619 with *pol31::KanMX4, pol32::hphMX, pol31-W417A* | pPOL31-W417 | this study |
| *GA-4630* | GA-4619 with *pol31::KanMX4, pol31-T415A* | pPOL31-T415 | this study |
| *GA-1981*  (W303) | *MATa, ade2-1 ,trp1-1 ,his3-11 ,-15, ura3-1, leu2-3, -112, can1-100, RAD5+* |  | (H.L. Klein) |
| *GA-6292* | GA-1981 with *Pol2-13Myc-KanMX, pol32::natMX* |  | this study |
| *GA-4780* | GA-4619 with *pol31::KanMX4, rev3::LEU2* | pRS316-POL31 | this study |
| *GA-9207* | GA-4619 with *pol31::KanMX4, pol31-W417A, rev3::natMX, pol32::hphMX,* | pPOL31-W417 | this study |
| *GA-9644* | GA-4619 with *pol32::hphMX4, rev3::natMX* |  | this study |
| *GA-9780* | GA-4619 with *pol31::KanMX4, rev3::LEU2, pol31-W417A* | pPOL31-W417 | this study |
| *GA-9664* | GA-4619 with *pol31::KanMX4, rad18::natMX* | pRS316-POL31 | this study |
| *GA-9665* | GA-4619 with *pol32::hphMX, rad18::natMX* |  | this study |
| *GA-9666* | GA-4619 with *pol31::KanMX4, pol31-W417A*, *pol32::hphMX, rad18::natMX* | pPOL31-W417 | this study |
| *GA-4817* | GA-4619 with *rad5::hphMX* |  | this study |
| *GA-9779* | GA-4619 with *pol31::KanMX, pol32::natMX, rad5::hphMX* | pRS316-POL31 | this study |
| *GA-9778* | GA-4619 with *pol31::KanMX, pol31-W417A , rad5::hphMX* | pPOL31-W417 | this study |
| *GA-9781* | GA-4619 with *pol31::KanMX, pol31-W417A , pol32::natMX, rad5::hphMX* | pPOL31-W417 | this study |
| GA-6123 | GA-1981 with *POL1-3HA-TRP1* |  | this study |
| GA-6290 | GA-6123 with *pol32::natMX* |  | this study |
| *GA-8997* | *MATa::DEL. HOcs::hisG ura3D851 trp1DEL.63 leu2DEL::KAN hmlDEL.::hisG hmrDEL::ADE3 ade3::GAL::HO can1DEL::UR:: HOcs::NAT, RA3::TRP1 (at*  *SUC2 locus in Chr IX)* |  | (J. Haber) |
| *GA-9133* | GA-8997 with *pol32::hphMX* |  | this study |
| *GA-9137* | GA-8997 with *pol31-T415A* |  | this study |
| *GA-9138* | GA-8997 with *pol31-W417A* |  | this study |
| *GA-4821* | GA-4619 with *mms2::hphMX4* |  | this study |
| *GA-9893* | GA-4619 with *mms2::hphMX4 pol32::hphMX4* |  | this study |
| *GA-9891* | GA-4619 *MATα* with *mms2::hphMX4 pol32::hphMX4 pol31::KanMX4 pol31-W417A* | pPOL31-W417 | this study |
| *GA-5050* | GA-1981 with *POL2-13Myc_KanMX* |  | this study |
| GA-6292 | GA-5050 with *POL2-13Myc_KanMX, pol32::NAT* |  | this study |
| *GA-10007* | GA-5050 with *POL2-13Myc-KanMX*, *pol31 ::URA3* | pPOL31-W417 | this study |
| *GA-10008* | GA-5050 with *POL2-13Myc-KanMX*, *pol31::URA3* | pPOL31-T415 | this study |
| *GA-4796* | GA-4619 with *POL3-9Myc_hphMX* |  | this study |
| *GA-4732* | *Mat a his3Δ1 leu2Δ0 met15Δ0 ura3Δ0* (BY4741) |  | K. Shirahige |
| *GA-4733* | GA4732 with *POL3-6HIS-3FLAG*-KanMX |  | D. Branzei |
| *GA-7625* | Mat α *ade2-1 ,trp1-1 ,his3-11 , -15, ura3-1, leu2-3, -112, can1-100 RAD5+ pol32::natMX* |  | this study |
| *GA-5998* | *mata::HOcsDEL::hisG ura3 DEL851 trp1DEL63 sup53DEL::leu2DEL::NATMX hmlDEL::hisG hmrDEL::ADE3 ade3::GAL10::HO can1,1-*  *1446::HOcs::HPH::DEL AVT2 yk1215c::leu2::hisG::can1DEL1-289* |  | J. Haber |
| *GA-5999* | GA-5998 with *pol32::KanMX* |  | J. Haber |
