# Supplementary material for: The stabilized Pol31–Pol3 interface counteracts Pol32 ablation with differential effects on repair
Source: Life Sci Alliance. 2021 Jul 5;4(9):e202101138. doi: 10.26508/lsa.202101138 (PMC8321694; doi:10.26508/lsa.202101138)
Supplement: Supplementary file 2 [file LSA-2021-01138_TableS2.docx]

**Table S2: Plasmids used in this study.** All plasmids used in this study are listed with relevant gene contained and source.

| Plasmid  No. | Plasmid Name | Source |
| --- | --- | --- |
| # *2731* | *pBL331 (pRS314-POL31 (-139 to +2044, ARS CEN TRP1)* | (Gerik et al.,1998) |
|  | *pRS316-POL31 (-139 to +2044, ARS CEN URA3)* | (Davoodi et al., 2006) |
|  | *pPOL31-D297 (-139 to +2044, ARS CEN URA3)* | (Davoodi et al., 2006) |
| # *3881* | *pPOL31-EF463 (-139 to +2044, ARS CEN TRP1)* | this study |
| # *2732* | *pPOL31-W417 (-139 to +2044, ARS CEN TRP1)* | this study |
| # *2736* | *pPOL31-T415 (-139 to +2044, ARS CEN TRP1)* | this study |
| # *359* | *pSH18-34 (2μ, URA3)* | (Golemis et al., 2011) |
| # *965* | *pGAL-LexA (2μ, HIS3)* | (Bjergbaek et al., 2005) |
| # *2686* | *pGAL-LexA-POL31 (aa1-487, 2μ, HIS3)* | this study |
| # *2856* | *pGAL-LexA-POL31-T415 (aa1-487, 2μ, HIS3)* | this study |
| # *2855* | *pGAL-LexA-POL31-W417 (aa1-487, 2μ, HIS3)* | this study |
| # *3816* | *pGAL-LexA-REV3 (aa1-1504, 2μ, HIS3)* | this study |
| # *1493* | *pJG47-HA (2μ, TRP1)* | this study |
| # *2693* | *pJG45-HA-POL3 (aa991-1097, 2μ, TRP1)* | this study |
| # *2694* | *pJG45-HA-POL3 (aa1032-1097, 2μ, TRP1)* | this study |
| # *3062* | *pJG47-HA-POL31 (aa1-487, 2μ, TRP1)* | this study |
| # *3063* | *pJG47-HA-POL31-T415 (aa1-487, 2μ, TRP1)* | this study |
| # *3064* | *pJG47-HA-POL31-W417 (aa1-487, 2μ, TRP1)* | this study |
| # *2949* | *pJG47-HA-PCNA (aa1-259, 2μ, TRP1)* | this study |
| # *3010* | *pJG47-HA-POL32 (aa1-350, 2μ, TRP1)* | this study |
| # *2905* | *pOPINM His-MBP-Pol3 (aa991-1097, T7-lacO, pUC-ori, Amp)* | this study |
| # *2906* | *pOPINM His-MBP-Pol3 (aa1032-1097, T7-lacO, pUC-ori, Amp)* | this study |
| # *3716* | *pRS305-POL31-T415 (aa1-487, LEU2)* | this study |
| # *3717* | *pRS305-POL31-W417 (aa1-487, LEU2)* | this study |
| #4072 | *pSPOT5 (CEN, LEU2)* | Chromotek |
| #4073 | *pSPOT5-POL3 (called pSPOT-Pol3)* | this study |
